# Adjuvant transarterial chemoembolization timing after radical resection is an independent prognostic factor for patients with hepatocellular carcinoma

**DOI:** 10.3389/fonc.2023.1129065

**Published:** 2023-03-09

**Authors:** Hongfa Sun, Hanlin Wang, Youpeng Wang, Wenqing Zhong, Yushan Meng, Ziqiang Lv, Weidong Guo, Bing Han

**Affiliations:** ^1^ Department of Hepatobiliary and Pancreatic Surgery, The Affiliated Hospital of Qingdao University, Qingdao, Shandong, China; ^2^ Department of Gastroenterology, The Affiliated Hospital of Qingdao University, Qingdao, Shandong, China

**Keywords:** HCC, TACE (transarterial chemoembolization), prognosis, timing, treatment

## Abstract

**Background:**

It has been reported that postoperative adjuvant TACE (PA-TACE) treatment decreases recurrence and significantly improves the survival of patients who undergo radical resection of hepatocellular carcinoma (HCC) with high-risk recurrence factors. However, when to perform PA-TACE has not been fully studied.

**Methods:**

We retrospectively collected the clinicopathologic characteristics of the patients with HCC between October 2013 and June 2020. The optimal cutoff value for PA-TACE time was determined based on the R package “maxstat”. Logistic regression and Cox regression analysis were used to determine the effect of the choice of PA-TACE timing on prognosis.

**Results:**

The analysis was performed on 789 patients with HCC, and 484 patients were finally involved and were divided into training cohort (378) and validation cohort (106). The PA-TACE timing was found to be associated with survival outcomes. Multivariate logistic analysis found independent predictors of the PA-TACE timing, including gender and history of HBV. Multivariate Cox analysis showed that Ki-67, tumor size, MVI and the PA-TACE timing were independent prognostic factors for RFS in HCC patients.

**Conclusions:**

Based on this study, HCC patients with high-risk recurrence factors can receive personalized assistance in undergoing PA-TACE treatment and improve their survival outcomes.

## Highlights

This retrospective analysis was performed on 789 patients with high-risk recurrence factors who had undergone radical hepatectomy for HCC. 484 patients were finally entered into the analysis and were divided into training cohort (378) and validation cohort (106). With the results of this study, the PA-TACE timing after radical resection is an independent prognostic factor for patients with HCC. HCC patients with high-risk recurrence factors can receive personalized assistance in undergoing PA-TACE treatment and improve their survival outcomes.

## Introduction

On a global scale, liver cancer is the fourth most common cause of cancer-related death and ranks sixth in terms of incidence (1). The most prevalent type of liver cancer, hepatocellular carcinoma (HCC), accounts for approximately 90% of all cases (2). There are curative treatments available for HCC patients, including resection, transplantation and ablation (3, 4). For most HCC cases, resection is the primary therapeutic option (4, 5). Despite this, tumor recurrence following hepatectomy remains a major hurdle in managing HCC effectively, with the 5-year recurrence rates reaching 60%-70% (6, 7). The conventional risk factors for recurrence include nonanatomical resection, tumor size, microvascular invasion (MVI), serum alpha-fetoprotein level (AFP) and multiple tumors (8–10). To improve the long-term prognosis of postoperative HCC, postoperative adjuvant treatments are urgently needed.

A variety of strategies employing adjuvant therapeutic modalities (both systemic and locoregional) have been proposed over the years, including transarterial chemoembolization (TACE), interferon (11), capecitabine (12), hepatic arterial infusion chemotherapy (13), sorafenib (14), immunotherapy and heparanase inhibitor PI-88 (15, 16), which have been proposed with varying degrees of success. When HCC is at an intermediate stage (BCLC), TACE is the first-line treatment recommended by the Barcelona Clinic Liver Cancer (BCLC) staging system. Meanwhile, TACE is regarded as a critical adjuvant therapy after radical resection in cases of HCC with high-risk recurrence factors to prevent recurrence. The effectiveness of postoperative adjuvant TACE (PA-TACE) in preventing recurrence and improving the prognosis of HCC has been established by a large number of studies (17–19). However, when to perform PA-TACE following radical hepatectomy and the factors affecting the PA-TACE timing have not been fully studied.

A retrospective analysis was conducted of the PA-TACE time, clinicopathological characteristics, and prognosis in HCC patients with high-risk recurrence factors. The recommended timing of PA-TACE was determined by using the optimal cutoff value method, and then the patients were divided into early and later TACE groups with significant prognostic differences. The potential factors affecting the PA-TACE timing were obtained by logistic regression analysis. Our final step was to incorporate the timing of PA-TACE into a multivariate Cox regression model and develop a prognostic nomogram to demonstrate that the PA-TACE timing was independently associated with the prognosis of HCC patients. Internal validation and comparison with conventional prognostic evaluation systems were also carried out. In our study, we assessed how the PA-TACE timing affects the prognosis of HCC patients, which provided recommendations for the PA-TACE timing after hepatectomy in HCC patients with high-risk recurrence factors and contributed to improving the prognosis of HCC.

## Methods

### Study population

**Figure 1:**
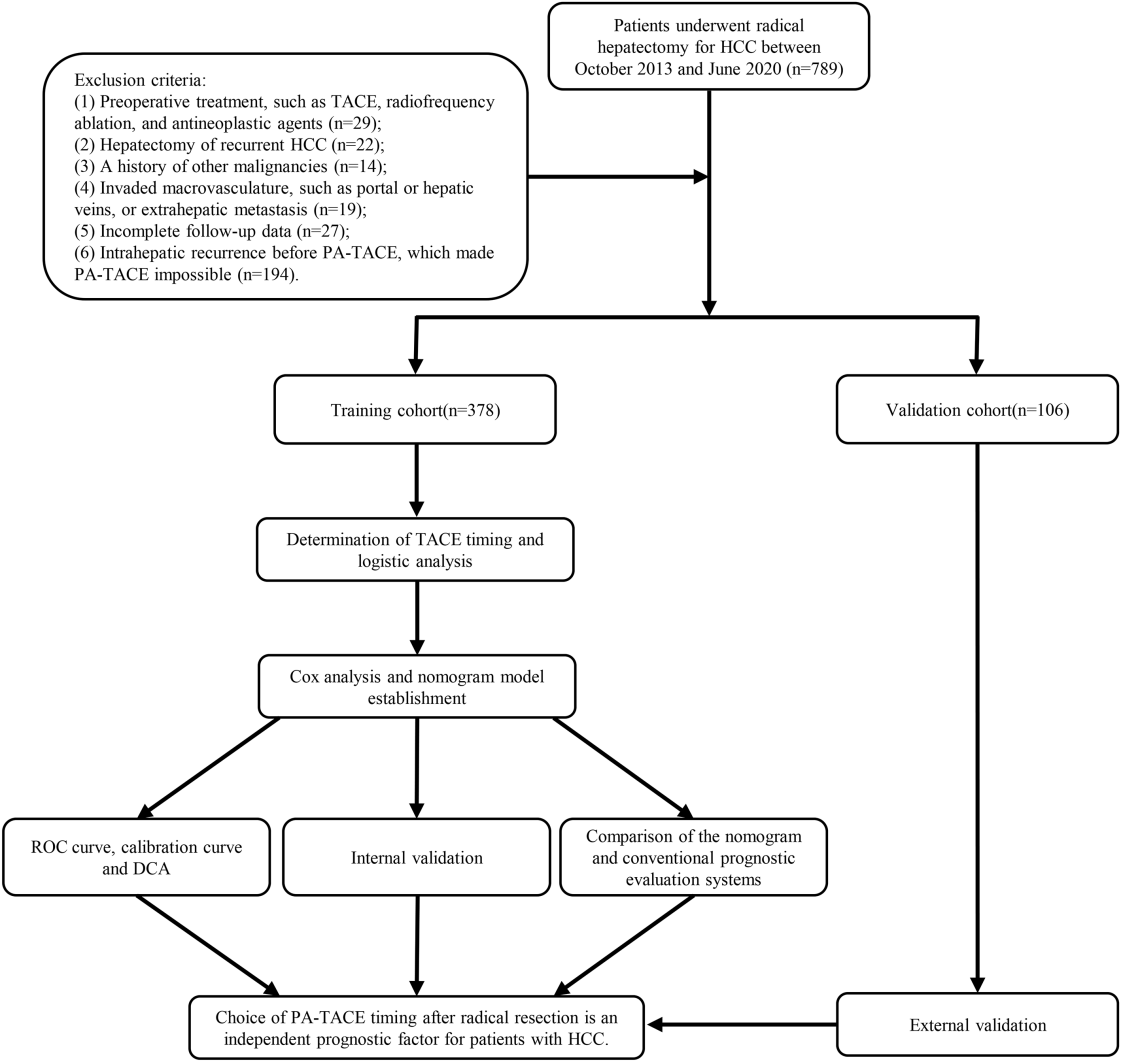
The flow chart of the study process.

We retrospectively identified consecutive patients with HCC who received radical hepatectomy as their primary therapy between October 2013 and June 2020 at the Affiliated Hospital of Qingdao University from our prospective database, and a diagnosis of HCC was confirmed by pathological reports. The inclusion criteria were as follows: (1) diagnosis of HCC by pathologic criteria; (2) >18 years and ≤80 years of age; (3) histopathologically confirmed HCC with a high risk of recurrence after resection and not receive targeted therapy and immunotherapy before recurrence.; (4) Child−Pugh classification A or B; (5) Eastern Cooperative Oncology Group performance score (ECOG PS) ≤2; and (6) R0 resection. The exclusion criteria were as follows: (1) preoperative treatment, such as TACE, radiofrequency ablation, and antineoplastic agents; (2) hepatectomy of recurrent HCC; (3) a history of other malignancies; (4) invaded macrovasculature, such as portal or hepatic veins, or extrahepatic metastasis; (5) incomplete follow-up data; and (6) intrahepatic recurrence before PA-TACE, which made PA-TACE impossible. The training cohort included 378 patients, and 244 were excluded. We set up a cohort of 167 patients from the Affiliated Hospital of Qingdao University between July 2019 and June 2020 as external validation. According to the inclusion criteria and exclusion criteria, 106 patients were included as the validation cohort. **Table 1** summarized the demographic and pathological characteristics of HCC patients. **Figure 1** shows the flow chart of the entire process.

**Table 1:** The demographic and pathological characteristics of the HCC patients.

	Training cohort (n=378)	Validation cohort (n=106)	P value
PA-TACE time (day)	41.03 (35.05,49.99)	41.91 (35.06,55.95)	0.646
TALT (40)	111 (29.37)	28 (26.42)	0.553
TAST (U/L)	25 (19,33.1)	23.55 (19.83,30)	0.479
TALB (g/L)	40.38 (36.74,46.15)	40.9 (36.65,47.7)	0.975
TTBIL (umol/L)	15.39 (12.2,20.71)	14.93 (11.87,19.7)	0.331
TAFP (ug/L)	6.09 (2.89,39.06)	5.92 (2.66,32.9)	0.817
TPT (t/s)	11.3 (10.4,12.3)	11.65 (10.6,12.7)	0.099
ALT (40)	146 (38.62)	36 (33.96)	0.381
AST (U/L)	29.15 (22.4,43)	28.1 (21,45.03)	0.810
ALB (g/L)	42.6 (38.79,48.47)	40.5 (37.6,49.3)	0.127
TBIL (umol/L)	16.9 (12.8,22.5)	16.07 (11.49,21.83)	0.484
AFP (20)	225 (59.52)	62 (58.49)	0.848
PT (t/s)	10.9 (10,11.73)	11.2 (10.1,11.9)	0.292
Ki-67	30 (20,50)	30 (20,40)	0.722
tumor size (cm)	4.2 (3,7)	5.1 (3.3,8)	0.219
tumor number	1 (1,1)	1 (1,1)	0.764
MVI	242 (64.02)	65 (61.32)	0.61
satellite lesions	54 (14.29)	18 (16.98)	0.491
high (cm)	1.7 (1.65,1.73)	1.7 (1.65,1.73)	0.456
weight (kg)	70 (62,76)	70 (64,80)	0.249
BMI	24.22 (22.02,26.35)	25.01 (22.84,27.02)	0.059
history of HBV	198 (52.38)	57 (53.77)	0.8
age	61 (54,68)	61 (55,67)	0.864
gender	311 (82.28)	90 (84.91)	0.525

PA-TACE, postoperative adjuvant TACE; ALT, alanine aminotransaminase; AST, aspartate aminotransferase; ALB, albumin; TBIL, total bilirubin; AFP, serum alpha- fetoprotein; PT, prothrombin time; MVI, microvascular invasion; BMI, body mass index. The indicators before PA-TACE were displayed as “T + indicators”, such as “TALT”.

Anonymized or confidential patient data were maintained, and patient privacy was protected. We followed the 1964 Helsinki Declaration and its later amendments or comparable ethical standards in all procedures. The Affiliated Hospital of Qingdao University Research Ethics Committee approved the study protocol, protocol code QYFY WZLL 27141.

### Clinicopathologic characteristics and definitions

A variety of clinical information of patients was collected through preoperative imaging examinations (including abdominal B-ultrasound and contrast-enhanced computer tomography (CT) or magnetic resonance imaging (MRI)), laboratory examinations (including routine blood test, blood biochemical examination, coagulation tests, tumor marker examination and hepatitis serology tests), pathologic features, tumor recurrence, and details of the follow-up or date of death. Age, gender, body mass index (BMI), and history of HBV were included in the basic data collected from patients. We collected clinical characteristics, including alanine aminotransaminase (ALT), aspartate aminotransferase (AST), albumin (ALB), total bilirubin (TBIL), serum alpha-fetoprotein (AFP) and prothrombin time (PT), before surgery and PA-TACE. The indicators before PA-TACE were displayed as “T + indicators”, such as “TALT”. Since TALT, ALT and AFP are extremely skewed distribution, we converted TALT and ALT into binary variables with the upper limit of normal value of 40U/L, and AFP into binary variables with the boundary of 20ug/L (20, 21), and conduct subsequent analysis. Ki-67, tumor size, tumor number, microvascular invasion (MVI) and satellite lesions were confirmed based on imaging examinations and pathologic examination. PA-TACE time was defined as the number of days from hepatectomy to PA-TACE. The optimal cutoff value for PA-TACE time was obtained through the “survminer” package’s surv_cutpoint() function of R software (22, 23). The clinicopathologic characteristics of the patients in training cohort are summarized in **Table 2**.

**Table 2:** The clinicopathologic characteristics of the HCC patients in training cohort.

Clinicopathologic characteristics	Total Patients	Early TACE group	Later TACE group	P value
RFS grouping	378	272	106	
gender	311 (82.28)	220 (80.88)	91 (85.85)	0.26
age (year)	61 (54,68)	60 (52.25,68)	64 (56.75,69)	0.03
PA-TACE time (day)	41.03 (35.05,49.99)	37.49 (33.93,41.99)	60.99 (52.94,75.06)	0.00
recurrence	232 (61.38)	157 (57.72)	75 (70.75)	0.02
RFS (year)	2.6 (0.75,3.64)	2.66 (0.85,3.70)	2.04 (0.55,3.60)	0.18
death	129 (34.13)	80 (29.41)	49 (46.23)	0.00
OS (year)	3.4 (2.62,5.03)	3.43 (2.66,5.03)	3.28 (2.29,5.04)	0.35
TALT (40)	111 (29.37)	91 (33.46)	20 (18.87)	0.01
TAST (U/L)	25 (19,33.1)	25.7 (19.03,33.30)	23.95 (17.9,32.98)	0.48
TALB (g/L)	40.38 (36.74,46.15)	40.3 (36.77,45.38)	41.1 (36.49,47.56)	0.40
TTBIL (umol/L)	15.39 (12.2,20.71)	15.2 (12.1,20.72)	15.94 (12.58,20.75)	0.53
TAFP (ug/L)	6.09 (2.89,39.06)	6.48 (2.98,42.56)	4.53 (2.48,33.41)	0.15
TPT (s)	11.3 (10.4,12.3)	11.3 (10.4,12.4)	11.25 (10.5,12.2)	0.56
ALT (40)	146 (38.62)	108 (39.71)	38 (35.85)	0.49
AST (U/L)	29.15 (22.4,43)	29.45 (22.27,42)	28.5 (22.38,43.3)	0.99
ALB (g/L)	42.6 (38.79,48.47)	42.66 (38.9,48.70)	42 (38.35,47.06)	0.31
TBIL (umol/L)	16.9 (12.8,22.50)	16.9 (13.1,22.28)	16.9 (11.96,22.73)	0.66
AFP (20)	225 (59.52)	162 (59.52)	63 (59.43)	0.98
PT (s)	10.9 (10,11.73)	10.9 (10.1,11.8)	10.8 (9.9,11.5)	0.24
Ki-67	30 (20,50)	30 (20,50)	30 (20,40)	0.75
tumor size (cm)	4.2 (3,7)	4.3 (2.85,7)	4.1 (3,7)	0.45
tumor number	1 (1,1)	1 (1,1)	1 (1,1)	0.35
MVI	242 (64.02)	185 (68.01)	57 (53.77)	0.01
satellite lesions	54 (14.29)	44 (16.18)	10 (9.43)	0.09
high (cm)	1.7 (1.65,1.73)	1.7 (1.65,1.74)	1.7 (1.65,1.73)	0.23
weight (kg)	70 (62,76)	70 (62,76)	69 (60,76.5)	0.35
BMI	24.22 (22.02,26.35)	24.24 (22.04,26.33)	23.88 (21.27,26.56)	0.58
history of HBV	198 (52.38)	127 (46.69)	71 (66.98)	0.00

RFS, disease free survival; OS, overall survival; PA-TACE, postoperative adjuvant TACE; ALT, alanine aminotransaminase; AST, aspartate aminotransferase; ALB, albumin; TBIL, total bilirubin; AFP, serum alpha- fetoprotein; PT, prothrombin time; MVI, microvascular invasion; BMI, body mass index. The indicators before PA-TACE were displayed as “T + indicators”, such as “TALT”.

All specimens were sampled according to the “Evidence-based Practice Guidelines for Standardized Pathological Diagnosis of Primary Liver Cancer in China: 2015 Update” using a 7-point baseline sampling protocol (24). MVI is a condition in which tumor cells are visible on microscopy in a portal vein, hepatic vein, or large capsular vessel of the surrounding hepatic tissue (25). The maximum diameter of the pathology specimen was used to define tumor size. Tumor number was classified as 1, 2 and 3. The term satellite lesions refers to microscopic HCC nodules separated from the tumor by at least 2 cm of uninvolved liver parenchyma and not included in tumor counts. The surgical specimens were examined by two senior pathologists with more than 10 years of hepatic pathology experience. For discordant cases, consensus was reached through discussion.

Based on tumor characteristics identified by pathology reports, we evaluated the risk of recurrence for resection and included patients with high-risk recurrence factors. When a single tumor with MVI, two or three tumors, or a single tumor larger than 5 cm without MVI was present, patients were considered to have high-risk recurrence factors (8, 9, 18, 19).

### Hepatectomy and TACE

Based on the Barcelona Clinic Liver Cancer (BCLC) staging system, we developed a treatment strategy for our patients. Child−Pugh classification and the indocyanine green (ICG) test were used to assess hepatectomy. Intraoperative sonography was used to determine the resection route. For inflow control of the liver during the operation, intermittent Pringle’s maneuver (15 min of clamping followed by 5 min of release) was applied in selected cases. We used an ultrasonic dissector or a pean-clamp for the transection of the liver parenchyma. The histologic examination showed that all patients had achieved R0 resection, which was defined as no residual tumor and a negative margin.

For all patients, the liver function, serum AFP level and contrast-enhanced CT or MRI of the abdomen were evaluated approximately one month after surgery. Following the exclusion of patients who were not suitable for PA-TACE, those with high-risk recurrence factors were recommended to undergo PA-TACE. Socioeconomic status and compliance with doctors played a major role in whether patients followed physicians’ recommendations. For this reason, we included patients up to 4 months after surgery in our study so we can study how the PA-TACE timing affected prognosis.

The Seldinger method was used to apply PA-TACE to the entire remnant liver. Any obvious tumor staining in the remnant liver was detected by hepatic angiography, computed tomography angiography, or both. An emulsion of lipiodol (5-10 mL) was applied after chemotherapeutic agents, including doxorubicin hydrochloride (10 mg), pirarubicin (THP), or pharmorubicin (20-40 mg), were administered slowly through the right and left hepatic arteries if tumor staining was not found. Based on body surface area and liver function, the dosage of lipiodol and doxorubicin was determined (26).

Suspicious imaging findings or biopsy-proven tumors were considered to be signs of recurrence (8). An evaluation of the therapeutic strategy was conducted once tumor recurrence was diagnosed based on tumor number, tumor location, liver function and general patient condition. Surgical reresection and ablation were used in the treatment with curative intent. The other treatment methods included TACE, targeted therapy, immunotherapy, etc.

### Follow-up

Recurrence-free survival (RFS) was the primary endpoint of this study; overall survival (OS) and safety of PA-TACE were the secondary endpoints. In the first two years after surgery, patients were followed up once every 2 months and then once every 3 months thereafter. Each follow-up visit included liver function assessments, tumor markers, and abdominal ultrasounds. The patients were scheduled for contrast-enhanced CT or MRI once every 6 months or when recurrence/metastasis was suspected. RFS was defined as the time from the date of operation to the first documented disease recurrence through independent radiological evaluation or liver biopsy, and or death by any cause, whichever occured first. OS was defined as the time from date of surgery to date of death regardless of the cause of death. We recorded adverse events (AEs) from the day of PA-TACE to the last day of follow-up. Using the Common Terminology Criteria for Adverse Events (CTCAE) version 4.0, the safety of PA-TACE was evaluated (27). All follow-up data were summarized as of the end of January 2022.

External validation of the nomogram model was performed through the validation cohort. Exploratory subgroup analyses of RFS were performed in patients by age (≤65 years vs. >65 years), gender (male vs. female) and BMI (normal vs. abnormal).

### Statistical analysis

SPSS software (version 25, IBM, New York, USA) was used for statistical analysis. The median (interquartile range) and the Mann−Whitney U test were performed for continuous variables with a skewed distribution, while the mean ± SD and t test were used for variables with a normal distribution. We compared categorical variables using the χ2 test or Fisher’s exact test and presented them as frequencies and proportions. The Kaplan−Meier method was used to plot survival curves, and the log-rank test was used to compare them. Univariate logistic regression analysis was performed. Then, to evaluate the risk factors affecting the PA-TACE timing, multivariate logistic regression analysis was conducted using the stepwise backward elimination procedure. The model fit was assessed with the Hosmer−Lemeshow goodness of fit test. Backward stepwise regression analysis was used to evaluate independent prognostic factors in univariate and multivariate Cox analyses.

We established a nomogram based on the results of multivariate Cox analysis using the package “rms” in R (version 4.1.2, Vienna, Austria). The receiver operating characteristic (ROC) curve and area under the ROC curve (AUC) were used to quantify the discriminatory ability of the nomogram (28). With the Kaplan−Meier method, the calibration curve was depicted to assess whether the nomogram prediction was in agreement with observed real outcomes. Bootstraps with 1000 resamples were used for the validation of the nomogram and calibration curve construction. As described by Vickers and colleagues (29), the R package “rmda” was used to perform decision curve analysis (DCA) based on the net benefit, which was used to evaluate the performance of the established nomogram in clinical decision-making. The C-index was used for external validation, internal validation and comparison of the nomogram and conventional prognostic evaluation systems. Statistical significance was defined as a P value <0.05 for two-tailed tests.

## Results

### Prognostic value of the PA-TACE time

**Figure 2:**
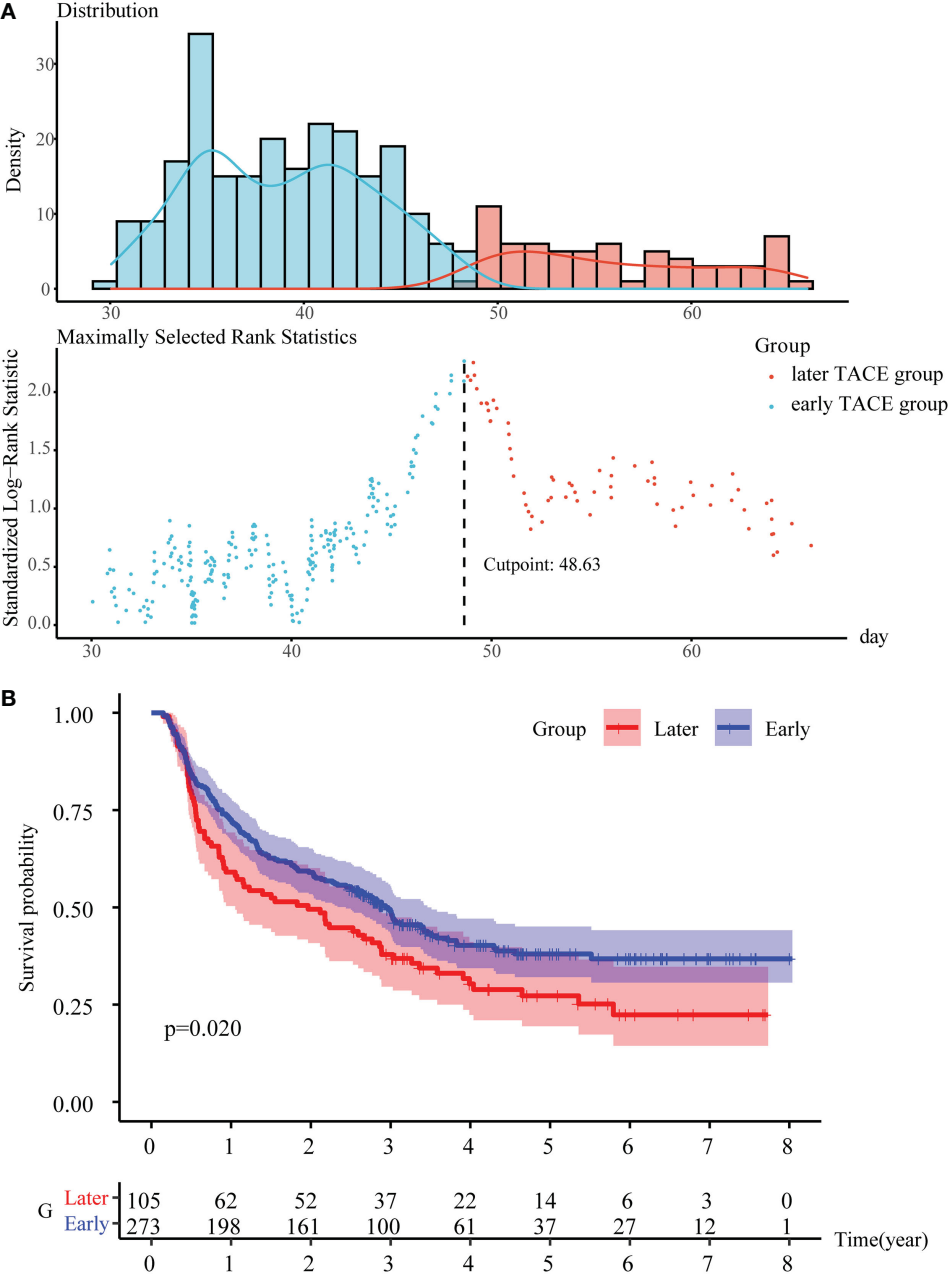
To determine the PA-TACE time cutoff values with significant prognostic differences. **(A)** Use the “survminer” package to get the optimal cutoff value for PA-TACE time. **(B)** K-M survival analysis of the early and later TACE groups for RFS. Abbreviations: PA-TACE, postoperative adjuvant TACE; RFS, disease-free survival.

The optimal cutoff value of the PA-TACE time was determined to be 48.63 days for RFS (**Figure 2A**), to confirm the effect of the PA-TACE timing on prognosis. We then divided the patients into early and later TACE groups based on the optimal cutoff value. The Kaplan−Meier analysis showed that the PA-TACE timing was a significant poor prognostic factor for RFS (P<0.05) (**Figure 2B**).

### Patients and clinicopathological characteristics

There was no significant difference in baseline characteristics between the training cohort and validation cohort (**Table 1**). A total of 789 HCC patients were treated at our center with radical (R0) partial hepatectomy. 484 qualified patients were enrolled in the final study and were divided into training cohort (378) and validation cohort (106). In training cohort: The median age of the patients was 61 (54-68) years, and 82.28% of them were male. The median time of PA-TACE was 41.03 (35.05-49.99) days. Postoperative pathologic examination confirmed that 242 (64.02%) patients had MVI, the median tumor diameter was 4.2 (3-7) cm, and 198 (52.38%) patients had HBV infection. As shown in **Table 2**, we divided the patients into early and later TACE groups based on the optimal cutoff value for RFS.

At the last follow-up in January 2022, the median RFS for all patients in training cohort was 2.6 (0.75-3.64) years, and the median OS was 3.4 (2.62-5.03) years. In the follow-up period, 232 (61.38%) patients relapsed, and 129 (34.13%) died, failing to reach the median survival. Compared to the late TACE group, the early TACE group had a significantly lower recurrence rate and mortality (57.72% vs. 70.75%, P<0.05; 29.41% vs. 46.23%, P<0.01). The RFS was 0.62 years longer in the early TACE group (2.66 years; 95% CI, 0.85-3.70 years) than in the later TACE group (2.04 years; 95% CI, 0.55-3.60 years). The OS was 0.15 years longer in the early TACE group (3.43 years; 95% CI, 2.66-5.03 years) than in the later TACE group (3.28 years; 95% CI, 2.29-5.04 years). The 1-, 3-, and 5-year RFS rates of the early TACE group were 72.1%, 48.8% and 37.7%, respectively; the 1-, 3-, and 5-year RFS rates of the later TACE group were 59.4%, 37.5% and 26.1%, respectively. In addition, the 1-, 3-, and 5-year OS rates of the early TACE group were 94.5%, 79.8% and 65.4%, respectively; the 1-, 3-, and 5-year OS rates of the later TACE group were 93.4%, 66.7% and 51.2%, respectively. In the validation cohort, the median RFS of HCC patients was 1.73 (0.93-2.07) years, and the median OS was 2.01 (1.71-2.22) years.

### Risk factors related to the PA-TACE timing

**Figure 3:**
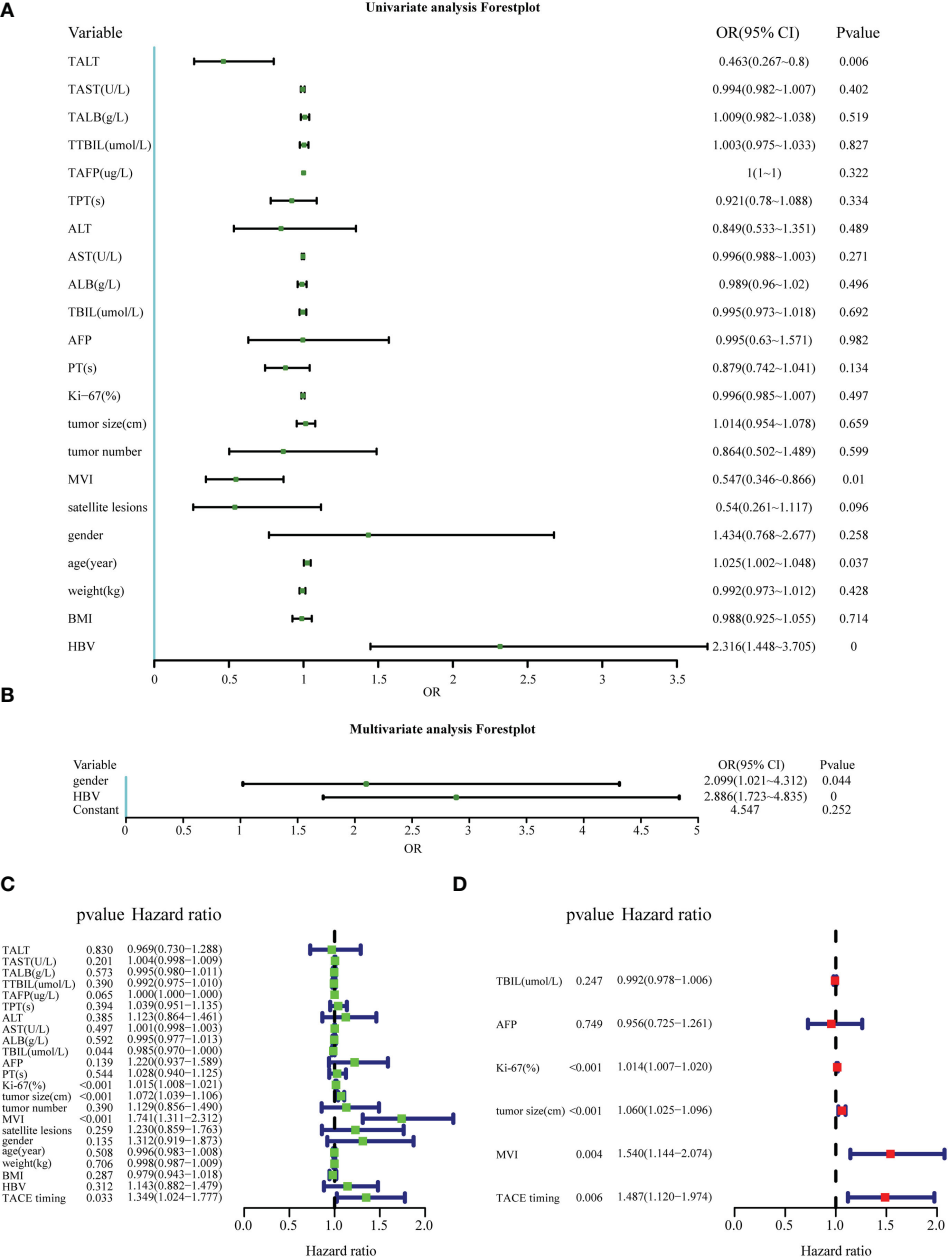
Factors affecting the PA-TACE timing and the prognosis of HCC. **(A, B)** Univariate and multivariate logistic analyses to evaluate the factors affecting the PA-TACE timing for RFS. **(C, D)** Univariate and multivariate Cox analyses to evaluate independent prognostic factors of HCC for RFS. ALT, alanine aminotransaminase; AST, aspartate aminotransferase; ALB, albumin; TBIL, total bilirubin; AFP, serum alpha- fetoprotein; PT, prothrombin time; MVI, microvascular invasion; BMI, body mass index. The indicators before PA-TACE were displayed as “T + indicators”, such as “TALT”.

Logistic regression analysis was conducted to identify risk factors associated with the PA-TACE timing. Regarding RFS, univariate logistic regression analysis revealed that TALT, ALT, AFP, MVI, satellite lesions, age and history of HBV may be risk factors associated with the PA-TACE timing (**Figure 3A**). In the RFS, multivariate logistic regression analysis found that independent predictors of the PA-TACE timing included gender(OR=2.099,95% CI,1.021-4.312, P<0.05) and history of HBV (OR=2.886, 95%CI,1.723-4.835, P<0.001) (**Figure 3B**). The values of Nagelkerke’s R2 were 0.224, while the results of the Hosmer−Lemeshow test were 0.762 in RFS analysis. These results showed that the overall model fit was good, with a median effect size.

### The PA-TACE timing included in cox analysis of prognosis of training cohort

Cox regression analysis was used to explore the effect of the PA-TACE timing on the prognosis of HCC. Univariate Cox analysis showed that TAFP, TBIL, AFP, KI-67, tumor size, MVI and the PA-TACE timing were risk factors for RFS in HCC patients (**Figures 3C, D**). Then, multivariate Cox analysis showed that Ki-67(HR=1.014, 95%CI,1.007-1.020, P<0.001), tumor size(HR=1.056, 95%CI,1.021-1.092, P=0.001), MVI(HR=1.503, 95%CI,1.114-2.028, P<0.01) and the PA-TACE timing (HR=1.515, 95%CI,1.139-2.015, P<0.01) were independent prognostic factors for RFS in HCC patients.

### Development and validation of the nomogram for the prognosis of HCC

**Figure 4:**
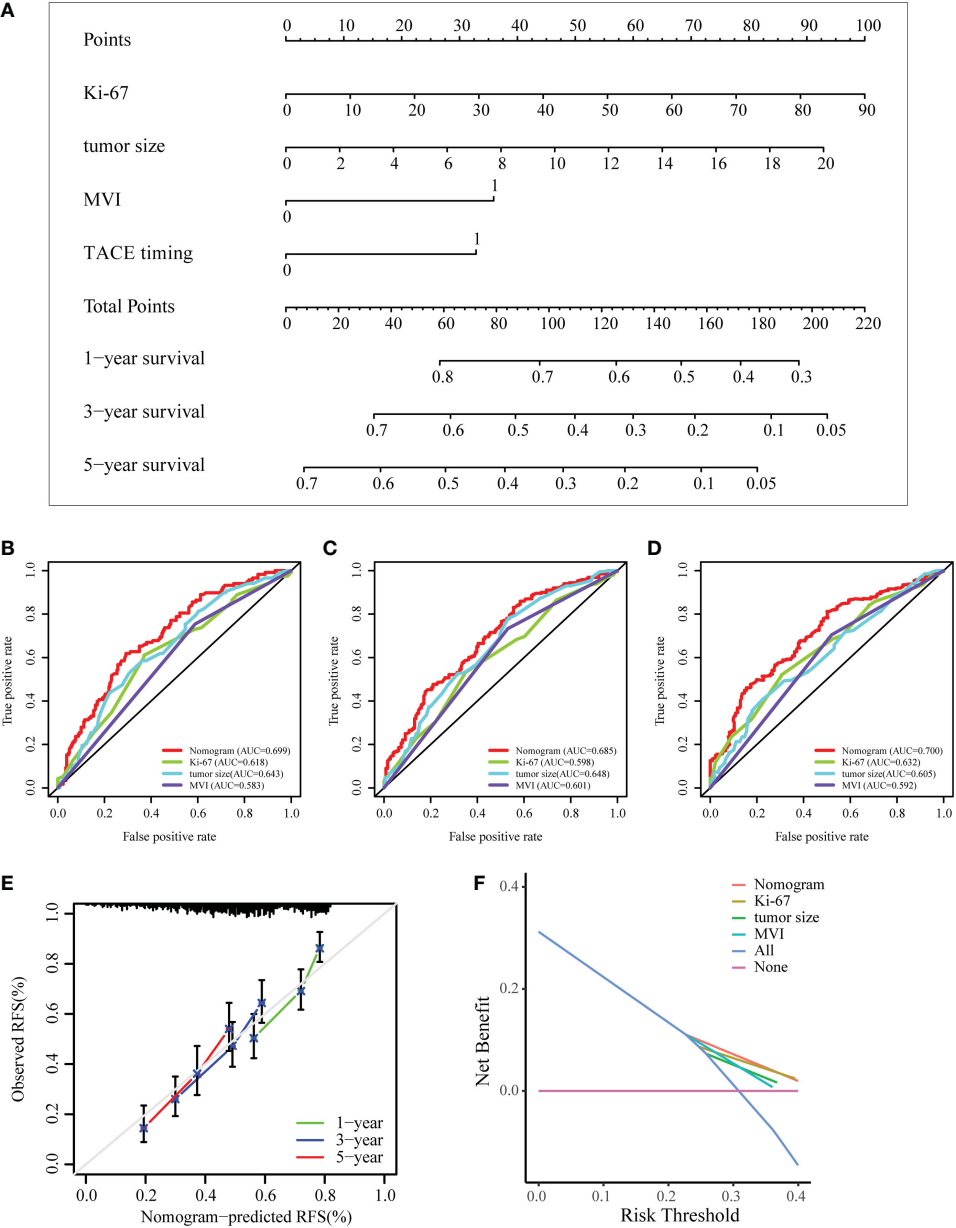
Development and validation of a nomogram for the prognosis of HCC. **(A)** Nomogram to evaluate the prognosis of HCC for RFS. **(B–D)** ROC curves of the model predicting the 1-, 3- and 5-year RFS of HCC patients. **(E, F)** The calibration plot and DCA of the nomogram for RFS. ROC, receiver operating characteristic curve; DCA, decision curve analysis; MVI, microvascular invasion.

To predict the prognosis of HCC patients, a nomogram was developed based on the training cohort that integrated the PA-TACE timing with significant clinical characteristics, such as Ki-67, tumor size and MVI, for RFS (**Figure 4A**). The ROC curve and AUC were calculated to evaluate and compare the discriminatory power of the nomogram model. The nomogram showed good predictive performance on the ROC curve. It was observed that the AUC values for 1-, 3- and 5-year RFS were 0.699, 0.685 and 0.700, respectively (**Figures 4B–D**). When comparing the constructed nomogram with the ideal model, the calibration plot showed good performance (**Figure 4E**). DCA also confirmed the predictive capacity of the nomogram (**Figure 4F**).

**Figure 5:**
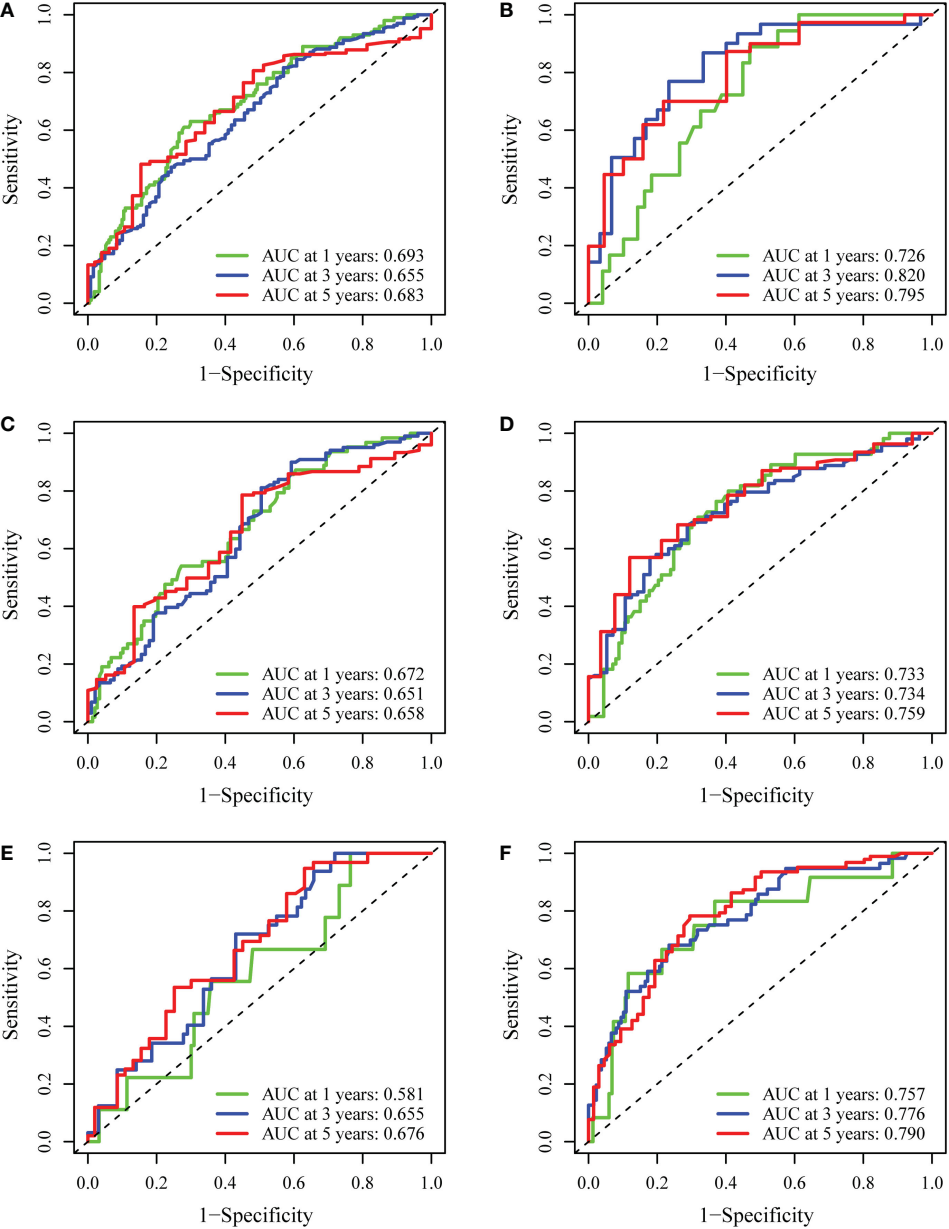
Internal validation of the nomogram model. **(A, B)** The predictive performance of the nomogram based on gender (male vs. female) for RFS. **(C, D)** The predictive performance of the nomogram based on age (>65 vs. ≤65) for RFS. **(E, F)** The predictive performance of the nomogram based on BMI (normal vs. abnormal) for RFS.

**Figure 6:**
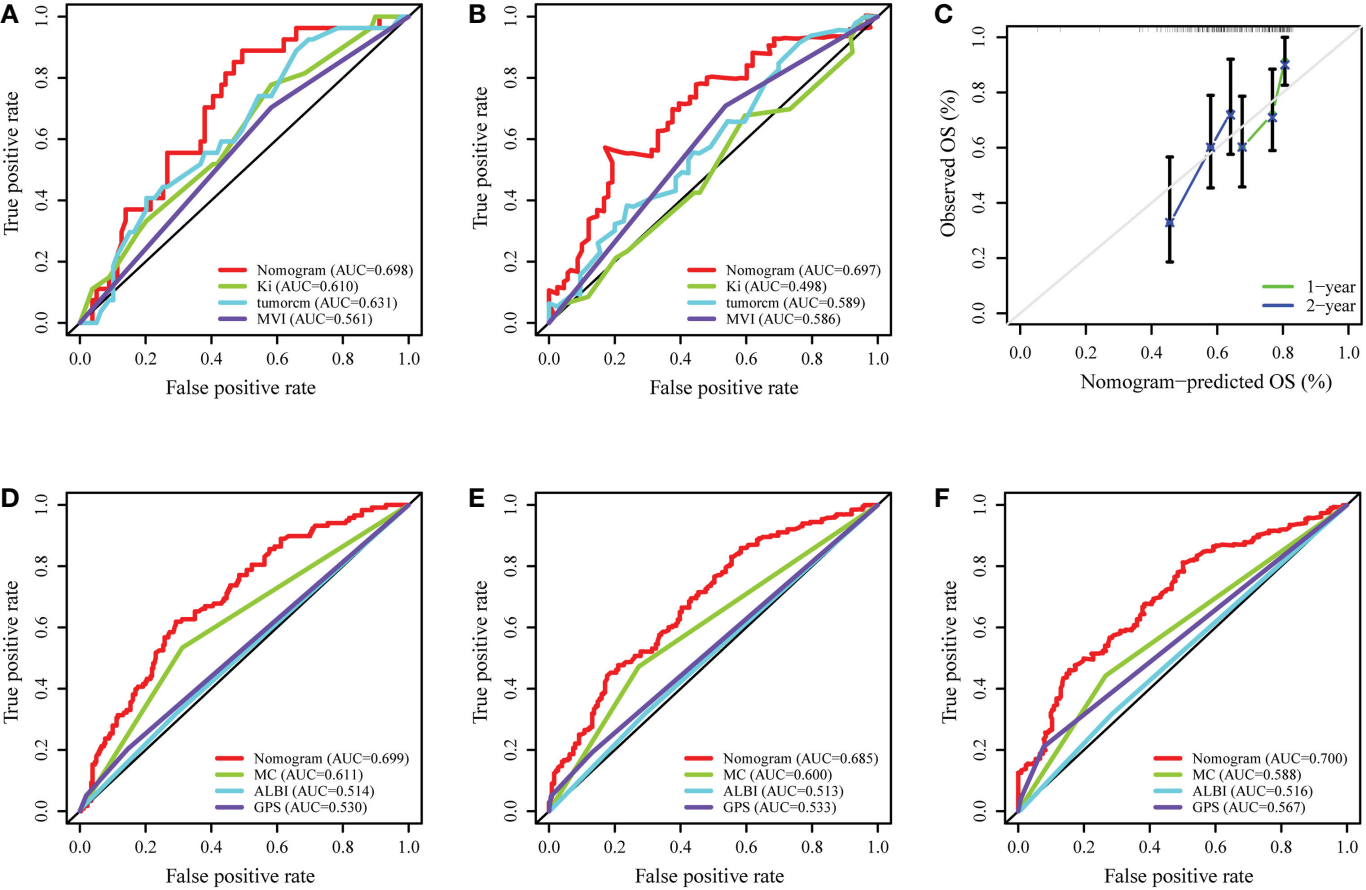
External validation and comparison of the nomogram model with the conventional prognostic evaluation systems. **(A, B)** ROC curves of the nomogram model predicting the 1- and 2-year RFS in validation cohort. **(C)** The calibration plot of the nomogram for RFS in validation cohort. **(D–F)** ROC curves of the nomogram model and the conventional prognostic evaluation systems predicting the 1-, 3- and 5-year RFS of HCC patients. MC, Milan criteria; ALBI, Albumin-Bilirubin grade; GPS, Glasgow Prognostic Score.

Internal validation was performed based on gender (male vs. female), age ( > 65 years vs. ≤65 years), and BMI (normal vs. abnormal) groupings to further validate the predictive power of the model for RFS. The results showed good predictive performance of the nomogram model for RFS in both the male and female subgroups (**Figures 5A, B**), the >65 and ≤65 age subgroups (**Figures 5C, D**), and the normal and abnormal BMI subgroups (**Figures 5E, F**). In the validation cohort, the 1-year and 2-year C-index of the nomogram were 0.698 and 0.697, respectively (**Figures 6A, B**). In addition, for the validation cohort, the 1-year and 2-year calibration plot of external validation of nomogram model performed well (**Figure 6C**). Furthermore, conventional prognostic evaluation systems, such as the Milan criteria (MC) (30), Albumin-Bilirubin (ALBI) grade and Glasgow Prognostic Score (GPS) (31, 32), were compared with the established nomogram to confirm which prognostic model was more reasonable and efficient. Our nomogram outperformed the conventional prognostic evaluation systems for RFS (**Figures 6D–F**) as measured by ROC curves of 1-, 3- and 5-year.

### Safety of TACE treatment and treatment after recurrence

**Table 3** summarizes the adverse events (AEs) related to PA-TACE in HCC patients. Overall, we found that most AEs were mild and manageable, and no toxicity-associated deaths occurred in this study. Nausea/vomiting (33.07%), pain (14.55%) and fever (13.49%) were the most common AEs. A few patients developed liver dysfunction (1.06%), leukopenia (0.53%) or thrombocytopenia (0.53%). No grade 3 or 4 AEs were observed based on the Common Terminology Criteria for Adverse Events (CTCAE) version 4.0.

**Table 3:** The adverse events (AEs) related to PA-TACE in HCC patients.

Adverse events	Total Patients (n=378)	Early TACE group (n=272)			Later TACE group (n=106)	
Grade	Grade 1-2	Grade 3-4	Grade 1-2	Grade 3-4	Grade 1-2	Grade 3-4
Nausea/vomiting	125 (33.07)	0	87 (31.99)	0	38 (35.85)	0
Pain	55 (14.55)	0	39 (14.34)	0	16 (15.09)	0
Fever	51 (13.49)	0	37 (13.60)	0	14 (13.21)	0
Leukopenia	2 (0.53)	0	0	0	2 (1.89)	0
Thrombocytopenia	2 (0.53)	0	0	0	2 (1.89)	0
Liver dysfunction	4 (1.06)	0	3 (1.10)	0	1 (0.94)	0

The patients in our study received subsequent antitumor therapies after recurrence, including TACE (40.52%), locoregional ablation (13.36%), hepatectomy (3.02%), targeted therapy and immunotherapy (14.66%) (**Table 4**). There were 34 patients who received targeted therapy and immunotherapy at the time of recurrence or suspected recurrence. We analyzed the effects of targeted therapy and immunotherapy on patients’ OS in **Table 5**. The analysis results showed that the OS of patients with targeted and immunotherapy was better than that of patients without targeted and immunotherapy, although there was no statistical difference (P>0.05).

**Table 4:** The subsequent antitumor therapies after recurrence.

	Total Patients (n=378)	Early TACE group (n=272)	Later TACE group (n=106)
recurrence	232 (61.38)	157 (57.72)	75 (70.75)
TACE	94 (40.52)	67 (42.68)	27 (36)
Locoregional ablation	31 (13.36)	21 (13.38)	10 (13.33)
Hepatectomy	7 (3.02)	5 (3.18)	2 (2.67)
Targeted therapy and immunotherapy	34 (14.66)	21 (13.38)	13 (17.33)
Conservative treatment	66 (28.45)	43 (27.39)	23 (30.67)

**Table 5:** Effects of targeted therapy and immunotherapy on OS in HCC patients after recurrence.

	Targeted therapy and immunotherapy (n=34)	No-targeted therapy and immunotherapy (n=344)	P value
Total	4.45 (2.83-5.53)	3.36 (2.60-4.92)	0.073
Early TACE group	4.36 (2.82-5.51)	3.38 (2.65-4.93)	0.179
Later TACE group	4.62 (2.75-5.62)	3.24 (2.22-4.90)	0.227

## Discussion

The 5-year recurrence rate after radical resection of HCC is as high as 60-70% (6, 7), which is an important reason for the poor survival outcomes of HCC patients (6, 33). A variety of adjuvant therapies, including interferon (11), capecitabine (12), hepatic arterial infusion chemotherapy (13), targeted therapy and immunotherapy (14, 15), have been reported with limited success. In the STORM trial of adjuvant sorafenib for HCC after resection or ablation, the primary endpoint of prolonged RFS was not reached (14). In recent years, a number of retrospective studies and prospective RCT trials have shown that PA-TACE treatment after radical resection of HCC can significantly reduce the tumor recurrence rate and improve the RFS and OS of patients with high-risk recurrence factors (18, 19, 34). As an important adjuvant therapy, TACE has formed a standardization of the technique to a certain extent through long-term development.

Conventional TACE (cTACE), which uses Lipiodol, and TACE with drug-eluting beads (DEB-TACE) are the two types of TACE techniques (35–38). The two TACE technologies are similar in tumor response and survival, while DEB-TACE has less systemic toxicity and adverse events (AEs) (37, 39). The chemotherapeutic agents used in TACE are generally doxorubicin or cisplatin (40, 41), and the choice of chemotherapeutic agent for TACE may not significantly affect the prognosis of patients (42). The fixed TACE schedule and tumor response guided retreatment (treatment on demand) strategy are both considered in retreatment decision-making, but fixed treatment strategies may have deleterious effects on liver function (43). The frequency of TACE also varies widely and is spaced as close as 2 weeks or as far as 8 weeks apart (41, 44). The STATE score, HAP score and ABCR score were developed to evaluate the criteria for the first and repeated TACE treatment of patients with intermediate stage HCC (BCLC) (45–47). These tools have shown limited predictive value. However, the PA-TACE timing after radical resection in patients with high-risk recurrence factors has not been reported. The PA-TACE time mentioned in various studies is approximately one month based on experience (17–19, 34). This study demonstrated that the PA-TACE timing after radical resection was an independent prognostic factor for HCC patients with high-risk recurrence factors.

In this retrospective study, the usual time for patients to undergo PA-TACE was approximately one month. Patients who undergo PA-TACE prematurely are prone to liver failure and other serious complications, as their liver function has not fully recovered, their albumin level is low, and infection has not been completely controlled (48–51). Furthermore, when major abdominal surgery is performed, such as hepatectomy, growth factors and proinflammatory cytokines (such as macrophage inflammatory protein-2, interleukin-6, and tumor necrosis factor alpha) are released that promote regeneration of the remaining liver tissue but may also inadvertently enhance the proliferation of these remaining tumor cells (52–54). Therefore, it is important to administer PA-TACE treatment before the tumor becomes difficult to control. Nevertheless, whether patients follow doctors’ recommendations is largely determined by their socioeconomic status and compliance with doctors. Interestingly, the phenomena found in our study can partially explain the above theory. ALT (TACE) in the early TACE group was higher than that in the later TACE group (31.2, 20.93-48 vs. 25, 19.08-36.18, P<0.01), while the age of the later TACE group was older than that of the early TACE group (60, 52.25-68 vs. 64, 56.75-69, P<0.05) in training cohort. One possible reason is that the elderly are less motivated with regard to disease treatment than relatively young people. The difference in the PA-TACE timing is thought to explain the different prognoses of HCC patients.

Using the optimal cutoff value method, we determined that the grouping cutoff values for RFS in the samples were 48.63 days, and the prognosis difference between the two groups was statistically significant. Logistic regression analysis showed that gender and history of HBV may be significant indicators to distinguish patients in early and later TACE groups. These indicators can provide guidance for the PA-TACE timing. We recommend that HCC patients with high-risk recurrence factors should undergo PA-TACE approximately one month after surgery and no later than 48.63 days. A large number of studies have confirmed that men have a greater risk of developing HCC than women worldwide, 2.35-fold more men were expected to die from HCC than women, and the same is true in China (55). However, it is not clear why men are more likely to develop HCC than women. The possible reasons are that the lower adiponectin levels found in men account for the increased incidence of HCC in men (56), and the different roles of the sex hormones (including androgens and estrogens and their corresponding receptors) and inflammatory mediators (IL-6, etc.) in the progression of HCC in men and women (57, 58). Due to the high incidence and mortality of HCC in male patients, we recommend that PA-TACE should particularly be performed in time after radical resection in male patients.

HBV was the first virus associated with the development of HCC and is the leading cause of HCC worldwide (59, 60). As a major aetiological factor, HBV infection changes the hepatic microenvironment, induces an inflammatory response, promotes angiogenesis and vascular invasion and affects the prognosis of HCC patients (61, 62). Similarly, we suggest that HCC patients with high-risk recurrence factors and HBV infection after radical resection should receive PA-TACE treatment in time under the guidance of doctors to obtain the best treatment outcome. Univariate and multivariate Cox regression analyses finally proved that the PA-TACE timing, Ki-67, tumor size and MVI were independent prognostic factors for HCC patients with high-risk recurrence factors. Ki-67 is a marker of proliferation. High-level Ki-67 expression in HCC tumors is associated with more rapid early recurrence (63, 64). Tumor size plays an important role in predicting HCC progression, and the risk of recurrence increases significantly as the tumor grows (65, 66). MVI is now widely used as a tool for assessing tumor aggressiveness and has been proven to be correlated with tumor recurrence and prognosis (67, 68). The nomogram model is more accurate in predicting RFS at 1, 3, and 5 years than individual clinicopathological risk factors. Additionally, the calibration curve and DCA results of the retest of the nomogram also showed a high level of prediction accuracy and good net benefit for RFS. Subgroup analysis suggested that the nomogram model provided predictive benefit to all the subpopulations. In external validation, the 1-year, 2-year calibration plot and ROC curves of nomogram model performed well. Furthermore, compared with conventional prognostic evaluation systems such as the Milan criteria (MC), albumin-bilirubin (ALBI) grade and Glasgow Prognostic Score (GPS), our nomogram still revealed good superiority.

Targeted therapy and immunotherapy after radical resection of HCC can improve the prognosis of patients (69, 70). There were 34 patients who received targeted therapy and immunotherapy at the time of recurrence or suspected recurrence. The results showed that targeted therapy and immunotherapy can improve the OS of HCC patients with recurrence, but further research was still needed.

There are several limitations to the present study. First, this was a retrospective, single-center study. A prospective, well-designed, multicenter, and randomized trial is required to validate the significance of the PA-TACE timing in HCC prognosis. Second, the majority of patients in this study (52.69%) had HBV-associated HCC. These results may not generalize to other causes of HCC. Third, all of the samples originated from China. Consequently, our findings may not be generalizable beyond Eastern Asia.

In conclusion, the PA-TACE timing is an independent factor affecting the prognosis of HCC patients with high-risk recurrence factors after radical resection. We have proposed that the recommended time for PA-TACE is about one month, no later than 48.63 days. Then, the gender and history of HBV are guiding indicators for PA-TACE. Moreover, based on multivariate Cox regression analysis, we established a nomogram model to predict the prognosis of HCC patients by combining the PA-TACE timing, Ki-67, tumor size and MVI. This study can provide personalized assistance for HCC patients with high-risk recurrence factors to undergo PA-TACE treatment and improve the survival outcomes of patients.

## Data availability statement

The raw data supporting the conclusions of this article will be made available by the authors, without undue reservation.

## Ethics statement

The studies involving human participants were reviewed and approved by The Affiliated Hospital of Qingdao University Research Ethics Committee approved the study protocol. The patients/participants provided their written informed consent to participate in this study.

## Author contributions

HS: Manuscript writing, research design, data collection, management and data analysis. HW: Research design, manuscript writing, data collection and data analysis. YW: Data collection, management and data analysis. WZ: Data collection and analysis. YM: Data collection and analysis. ZL: Data collection and analysis. WG: Project development and manuscript editing. BH: Project development, research design, and manuscript editing. All authors contributed to the article and approved the submitted version.
